# Long‐term strength and functional status in inclusion body myositis and identification of trajectory subgroups

**DOI:** 10.1002/mus.26859

**Published:** 2020-03-13

**Authors:** Alexander G. S. Oldroyd, James B. Lilleker, Jacob Williams, Hector Chinoy, James A. L. Miller

**Affiliations:** ^1^ NIHR Manchester Biomedical Research Centre, Manchester University NHS Foundation Trust, Manchester Academic Health Science Centre Manchester United Kingdom; ^2^ Centre for Musculoskeletal Research, University of Manchester, Manchester Academic Health Science Centre Manchester United Kingdom; ^3^ Centre for Epidemiology Versus Arthritis University of Manchester Manchester United Kingdom; ^4^ Department of Rheumatology Salford Royal NHS Foundation Trust Salford United Kingdom; ^5^ Manchester Centre for Clinical Neurosciences Salford Royal NHS Foundation Trust, Manchester Academic Health Sciences Centre United Kingdom; ^6^ Manchester Medical School University of Manchester Manchester United Kingdom; ^7^ Department of Neurology Royal Victoria Hospitals, The Newcastle upon Tyne Hospitals NHS Foundation Trust Queen Victoria Road, Newcastle United Kingdom

**Keywords:** inclusion body myositis, dynamometry, functional status, longitudinal modeling, muscle strength, myositis, trial outcomes

## Abstract

**Introduction:**

Objective information on longitudinal disease progression in inclusion body myositis (IBM) is lacking.

**Methods:**

Longitudinal dynamometry and functional status data were collated from a cohort of IBM patients. Annual change was calculated by means of linear modeling. Trajectories of change in grip, knee extension, IBM Functional Rating Scale (IBM‐FRS) and Neuromuscular Symptom Score (NSS) were identified by means of latent growth mixture modeling.

**Results:**

Data were collated from 75 IBM patients (348 person‐years follow‐up). Annual strength loss was greatest for pinch (−10%) and knee extension (−4%). Functional deterioration was greatest for males. Three distinct trajectory groups were identified. Rapid deterioration trajectory for grip strength was associated with younger diagnosis age. Rapid deterioration for knee extension strength was associated with older age of diagnosis.

**Discussion:**

This study has quantified strength change in IBM and identified distinct trajectory groups, which will aid prognostication and stratification for inclusion into future clinical trials.

## Abstract 2

See editorial on pages 7–9 in this issue.

**Abbreviations**
6‐MWD: 6‐Minute Walk Distance
anti‐CN1A: anti‐cytosolic‐5′‐nucleotidase 1A
ELISA: enzyme‐linked immunosorbent assay
FDA: United States Food and Drug Administration
GMM: growth mixture model
HRA: Health Research Authority
IBM: inclusion body myositis
IBM‐FRS: inclusion body myositis Functional Rating Score
IIM: idiopathic inflammatory myopathies
IQR: interquartile range
MRC: Medical Research Council
NSS: Neuromuscular Symptom Score
OD450: optical densities at 450 nm

## 1 INTRODUCTION

Sporadic inclusion body myositis (IBM) is a rare muscle disorder usually affecting those over age 50 years, characterized by slowly progressive weakness of the distal upper limbs and proximal lower limbs.

Unlike other idiopathic inflammatory myopathy (IIM) subtypes, patients with IBM do not respond clinically to immunosuppressive therapy and several factors conspire to make translational research endeavors challenging. The rarity of IBM and slow rate of disease progression make conduct of well‐powered clinical studies difficult. The usefulness of IBM clinical trial outcome measures is limited due to their inability to detect early improvement or stabilization of function that might reduce the required trial duration. Finally, our incomplete understanding of disease etiopathogenesis means that optimal drug targets are unknown.

In IBM, disease heterogeneity makes prognostication in clinical practice difficult and can affect interpretation of clinical trial results. The rate of disease progression can vary between IBM patients, although explanations for this are unclear.^1^ Of note, positivity for the autoantibody against anti‐cytosolic‐5′‐nucleotidase 1A (anti‐CN1A) is associated with a more severe disease phenotype.^2^, ^3^ In one cross‐sectional study, anti‐CN1A antibody positivity was associated with increasing levels of weakness as measured by clinical examination, but not by dynamometry (grip and pinch).^3^ However, clinical trials to date in IBM have not stratified participants according to anti‐CN1A serotype and longitudinal data examining the rate of change relative to anti‐CN1A serotype are not available.

Dynamometry is a simple technique for quantifying muscle strength and has been used extensively to monitor patients with neuromuscular disorders,^4^ and as a clinical trial outcome measure.^5^, ^6^ Measurements can easily be performed in the clinical setting, and the selective pattern of weakness in IBM lends itself to the use of dynamometry, as relatively few muscle groups require testing to capture disease severity. Several studies have attempted to capture longitudinal muscle strength data in IBM, although included numbers are usually small with short follow‐up.^1^, ^7^, ^8^, ^9^

It remains unclear whether dynamometry can reliably monitor disease progression in IBM, how data could be used in clinical practice to individualize patient care or in research studies as a trial outcome measure, and how these changes correlate with other measures of IBM disease severity, particularly those assessing functional impact.

This study aimed to: (a) quantify the yearly rate of dynamometry‐measured strength loss across multiple muscle groups in a large IBM cohort, and (b) identify distinct trajectories of strength and functional change over time, with the aim of individualizing patient care and providing useful data for future clinical trial design.

## 2 METHODS

Adult IBM patients fulfilling the Medical Research Council (MRC) 2010 possible,^10^ clinically defined or pathologically defined criteria reviewed between 2004 and 2015 at a single specialist neuromuscular clinic (Royal Victoria Infirmary, Newcastle‐upon‐Tyne, UK) were included in this retrospective analysis. Data were collected for the purposes of routine clinical care. Given this context, and after consultation with the Health Research Authority (HRA) using the HRA decision tools website,^11^ conduct of this study proceeded without additional ethical authorization. Anonymized demographic and clinical information, including anti‐CN1A autoantibody status, were collated.

For determination of anti‐CN1A autoantibody status, sera were analyzed in the Department of Biomolecular Chemistry in Nijmegen as part of a previous research project from which a subset of patients were recruited.^2^ Briefly, enzyme‐linked immunosorbent assay (ELISA) was performed with the three synthetic peptides containing CN1A auto‐epitopes previously identified by overlapping peptide microarray analyses.^12^ Signals were quantified by determining optical densities at 450 nm (OD450) using methods previously described and defined as seropositive if the OD450 value was greater than or equal to the established cut‐off value for the corresponding peptide.^13^

Patients were followed prospectively according to clinical need from their time of presentation. Per routine clinical care, quantitative muscle strength measurements using a single CITEC dynamometer (Haren, The Netherlands) were obtained at each clinic visit, which occurred at variable intervals. This was performed by dedicated neurophysiotherapists who received training provided by local muscle trials experts, based at the John Walton Muscular Dystrophy Centre (Newcastle, UK), to minimize inter‐rater variability. The force recorded was that required by the examiner to overcome maximum force exerted by the subject (break point), or the maximum exerted force if unable to reach break point. The measurement was repeated until two values within 10% were recorded, and the mean then calculated. The strength of up to 11 different left‐sided movements (6 upper‐limb and 5 lower‐limb) were measured at each visit (see Table 2). Patients also completed two questionnaires at each visit: the Neuromuscular Symptom Score (NSS)^14^ and IBM‐Functional Rating Score (IBM‐FRS).^15^

The mean baseline strength of each movement was calculated for the female and male cohorts and compared against reference values of healthy controls.^16^ Reference values for pinch strength were not available; therefore, no comparison against baseline measured values could be carried out. Each patient's first strength measurement was designated as their baseline. The difference of each subsequent strength measurement compared with the baseline was calculated. The percentage change from baseline was subsequently calculated for each strength measurement. Each movement was considered separately. The yearly rate of strength change for each movement was calculated across the cohort by means of simple linear regression. The yearly rates of NSS and IBM‐FRS change were also calculated.

Latent growth mixture modeling (GMM) is a statistical technique that separates a cohort into different groups, depending on longitudinal variables.^17^ This methodology has not previously been applied to longitudinal dynamometry data in IBM. Identification of separate trajectory groups within the cohort was carried out by means of latent GMM for grip, knee extension, IBM‐FRS, and NSS. Groups were identified according to strength/score change over time. Grip and knee extension were selected post hoc due to their known particular predilection for strength loss in IBM and high number of available strength measurements.^18^ Pinch was not included in GMM analysis due to sufficient longitudinal data only being available in 25 patients. Each study participant was assigned to a single group for grip, knee extension, IBM‐FRS, and NSS separately. The number of trajectory groups within the cohort that provided the best fit was identified by standard model comparison techniques: Bayesian Information Criterion and Akaike Information Criterion.^19^ Baseline demographics (age of IBM onset, gender) and anti‐CN1A autoantibody positivity were compared between trajectory groups using statistical inference tests: Wilcoxon signed rank test when comparing continuous variables and Kruskal‐Wallis test comparing proportions between three or more groups. All analysis was carried out using the statistical program R and the LCMM package.^20^, ^21^

## 3 RESULTS

Data from 75 IBM patients were analyzed with a total of 348 person‐years and a median 4 years (interquartile range [IQR] 3, 6) follow‐up (Table 1). Fifteen (20%) patients fulfilled the MRC possible criteria, 55 (73%) the clinically defined criteria, and 5 (7%) fulfilled the pathologically defined criteria. Overall, 5684 measurements were carried out (see the Supporting Information Material, which is available online). Mean baseline strength values, reference strength values and calculated differences are displayed in Table 2. Baseline strength of all movements was lower compared with reference values for both the female and male cohorts. The difference was proportionally greatest for knee extension and ankle dorsiflexion in both cohorts. The difference was proportionally lowest for shoulder abduction in both cohorts. The estimated yearly rate of change in strength for each movement, NSS, and IBM‐FRS are displayed in Table 3. Considering the whole cohort, strength loss over time was demonstrated in all measured movements, apart from elbow flexion and wrist extension, which were spared. Pinch demonstrated the greatest degree of yearly strength change, followed by knee extension.

**Table 1:** Cohort demographics

Variable	Value
Number	75
Number female (%)	35 (46.7)
Median age at time of diagnosis / years (range)	68.8 (52.9, 85.6)
Median time from diagnosis to first strength measurement / years (IQR)	0.4 (0.1, 0.8)
Median years of strength measurement follow up / years (IQR)	4.3 (2.6, 6.2)
Number tested for anti‐CN1A positivity (%)	32 (42.7)
Number anti‐CN1A positive (%)	13 (40.6)

**Table 2:** Reference and measured strength values for each movement

	Reference mean muscle strength / Newtons (SD)		Baseline mean muscle strength / Newtons (SD)		Difference between measured and reference strength values (%)	
Movement	Female	Male	Female	Male	Female cohort (*n* = 35)	Male cohort (*n* = 40)
Shoulder abduction	110.9 (37.3)	206.0 (62.8)	89.3 (33.4)	137.3 (56.6)	−21.6 (−19.5)	−68.7 (−33.3)
Elbow flexion	166.8 (35.3)	274.7 (56.9)	79.1 (41.4)	132.7 (55.9)	−87.7 (−52.6)	−142 (−51.7)
Elbow extension	100.1 (21.6)	166.8 (38.3)	56.8 (22.0)	98.5 (39.8)	−43.3 (−43.3)	−68.3 (−40.9)
Wrist extension	97.1 (26.5)	167.8 (42.2)	48.1 (26.0)	68.1 (29.7)	−49 (−50.5)	−99.7 (−59.4)
Grip^a^	70.0 (20.0)	100.0 (20.0)	28.4 (11.4)	43.8 (30.1)	−41.6 (−59.4)	−56.2 (−56.2)
Pinch^b^			35.2 (8.4)	53.8 (31.0)		
Hip flexion	246.2 (85.3)	364.9 (111.8)	92.1 (30.2)	122.2 (53.3)	−154.1 (−62.6)	−242.7 (−66.5)
Hip abduction	188.4 (60.8)	312.0 (87.3)	139.4 (52.1)	183.1 (84.2)	−49 (−26.0)	−128.9 (−41.3)
Knee extension	318.8 (98.1)	520.9 (141.3)	78.2 (39.5)	130.6 (71.5)	−240.6 (−75.5)	−390.3 (−74.9)
Knee flexion	185.4 (54.9)	290.4 (74.6)	125.5 (30.9)	147.0 (33.1)	−59.9 (−32.3)	−143.4 (−49.4)
Ankle dorsiflexion	461.1 (185.4)	709.3 (230.5)	136.6 (51.6)	132.2 (63.8)	−324.5 (−70.4)	−577.1 (−81.4)

a Values in Newtons/m^2^.

b Reference strength values were not available for pinch.

Abbreviation: SD, standard deviation.

**Table 3:** Yearly rate of change of strength and functional assessment tool results

	Estimated yearly percentage change ‐ % (95% CI)		
Measurement	Total cohort (*n* = 75)	Male cohort (*n* = 40)	Female cohort (*n* = 35)
Shoulder abduction	−4.0 (−4.2, −3.8)	−4.0 (−4.2, −3.8)	−4.0 (−4.3, −3.7)
Elbow flexion	0.1 (−0.7, 0.8)	−0.1 (−0.7, −0.8)	−0.1 (−0.4, 0.3)
Elbow extension	−2.1 (−2.3, −1.9)	−2.0 (−2.7, −1.3)	−2.3 (−3.1, −1.6)
Wrist extension	1.6 (0.1, 3.1)	2.0 (0.7, 3.3)	−5.7 (−9.6, −1.8)
Grip	−2.4 (−3.6, −1.3)	−2.7 (−4.3, −1.2)	−7.1 (−7.3, −6.8)
Pinch	−10.1 (−11.0, −9.1)	−11.3 (−13.3, −9.7)	−5.4 (−7.6, −3.3)
Hip flexion	−4.1 (−4.4, −3.8)	−2.3 (−3.1, −1.5)	−4.8 (−5.1, −4.5)
Hip abduction	−3.8 (−4.0, −3.6)	−6.0 (−6.5, −5.6)	−3.3 (−3.5, −3.1)
Knee extension	−4.2 (−5.0, −3.4)	−6.3 (−7.0, −5.6)	−5.1 (−5.6, −4.6)
Knee flexion	−1.8 (−2.9, −0.7)	4.0 (1.9, 6.2)	−1.2 (−5.3, 2.9)
Ankle dorsiflexion	−2.5 (−2.8, −2.3)	−5.6 (−5.6, −5.4)	−1.5 (−1.8, −1.1)
NSS	−1.3 (−1.5, −1.2)	−1.6 (−1.8, −1.5)	−1.0 (−1.2, −0.8)
IBM‐FRS	−1.3 (−1.6, −0.9)	−4.0 (−4.2, −3.8)	−1.1 (−1.4, −0.7)

Abbreviations: CI, confidence interval, n, number.

Patterns of hand involvement varied between the male and female cohorts, with males demonstrating greatest rate of loss of pinch strength, whereas females demonstrated the greatest rate of loss of grip strength. Of the lower limb movements, knee extension demonstrated the greatest rate of loss of muscle strength in both male and female cohorts. Ankle dorsiflexion demonstrated strength loss in both cohorts; however, this was greater for the male cohort, compared with the female cohort.

Reduction of both the NSS and IBM‐FRS were observed over time. For both, the rate of reduction was more pronounced in the male cohorts, and was particularly apparent for the IBM‐FRS.

**Figure 1:**
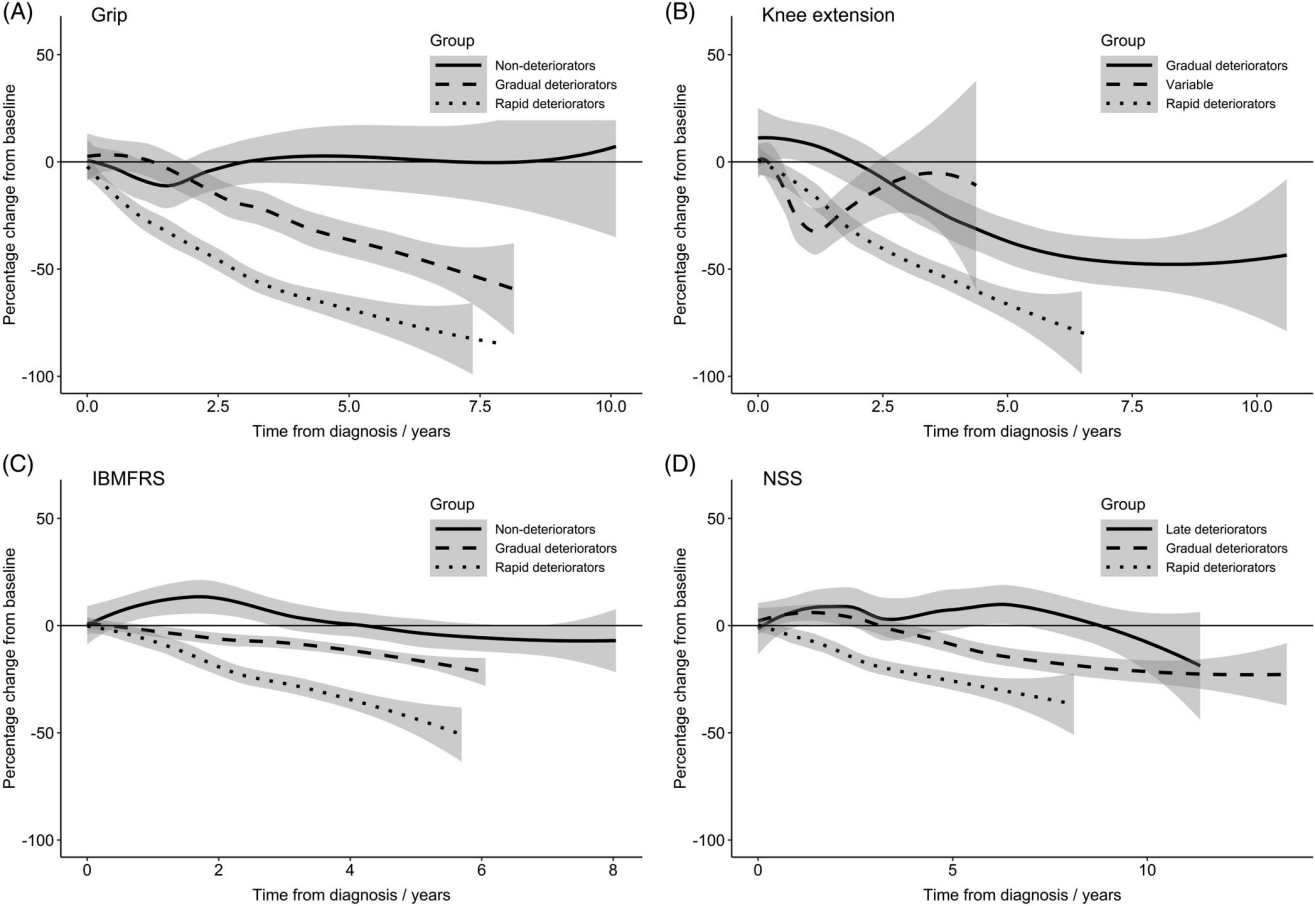
Longitudinal trajectories of grip (A) and knee extension (B) strength, IBM‐FRS (C), and NSS (D)

Identification of latent groups for grip, knee extension, IBM‐FRS and NSS was performed, with best fit for models with three groups in each case. The estimated trajectory of each identified group is displayed in Table 4 and Figure 1.

**Table 4:** Strength change trajectory groups identified for grip, knee extension, IBM‐FRS, and NSS

Variable	Group	Number (%)	Number female (%)	Median age at diagnosis / years (IQR)	Number tested for CN1A (%)	Number CN1A positive (%)	Median baseline value a (IQR)
Grip	Nondeteriorators	37 (60)	15 (41)	70 (63, 75)^b^	14 (38)	6 (43)	31 (23, 38)
	Gradual deteriorators	9 (15)	6 (67)	69 (59, 70)^b^	7 (78)	1 (14)	37 (25, 51)
	Rapid deteriorators	16 (26)	7 (44)	64 (58, 69)^b^	7 (44)	5 (71)	35 (29, 45)
Knee extension	Gradual deteriorators	6 (9)	2 (33)	64 (61, 68)^b^	6 (100)	3 (50)	174 (82, 192) b
	Variable	38 (59)	18 (47)	67 (60, 75)^b^	14 (37)	6 (44)	78 (47, 137) b
	Rapid deteriorators	20 (31)	9 (45)	69 (66, 72)^b^	10 (50)	4 (40)	74 (61, 124) b
IBM‐FRS	Nondeteriorators	3 (6)	3 (100)	66 (62, 67)	2 (67)	1 (50)	34 (32, 35)
	Gradual deteriorators	16 (31)	5 (31)	67 (60, 71)	6 (38)	2 (30)	25 (23, 33)
	Rapid deteriorators	32 (63)	15 (47)	69 (62, 73)	13 (41)	4 (31)	29 (26, 33)
NSS	Late deteriorators	6 (8)	3 (50)	60 (58, 68)	4 (67)	2 (50)	42 (29, 47)
	Gradual deteriorators	7 (10)	2 (29)	67 (66, 68)	5 (71)	1 (20)	42 (34, 48)
	Rapid deteriorators	58 (82)	28 (48)	69 (62, 75)	20 (34)	7 (35)	43 (33, 48)

a Strength values are presented in Newtons.

b P < 0.01 (continuous variables were compared using the Wilcoxon signed rank test and Kruskal‐Wallis test comparing proportions between three groups).

For grip, the largest proportion of the cohort demonstrated gradual strength loss, while smaller proportions followed “rapid deteriorator” and “gradual deteriorator” trajectories. Median baseline strength measurements within each group were similar. Age at time of IBM diagnosis significantly varied across the three groups, with the “rapid deteriorators” demonstrating the youngest age and “nondeteriorators” demonstrating the oldest age. Where tested, anti‐CN1A antibody positivity varied between each of the three identified groups, with the majority of the “rapid deteriorator” group being anti‐CN1A positive, compared with a minority in the “nondeteriorator” group, although no significant difference was identified.

For knee extension, the majority followed a “variable” trajectory, with no overall strength deterioration after 4 years of follow‐up. The median baseline strength measurement was substantially higher for the “gradual deteriorator” group and similar for both the “variable” and “rapid deteriorator” groups. Age significantly varied across the three groups, with younger patients belonging to the “gradual deteriorator” group and older patients belonging to the “rapid deteriorator” group.

For IBMFRS, the majority followed a “rapid deteriorator” trajectory and only a minority demonstrated no deterioration. The “rapid deteriorator” IBM‐FRS trajectory group demonstrated the oldest age, followed by the “gradual deteriorator” and “nondeteriorator” groups.

For NSS, the majority followed a rapidly deteriorating NSS trajectory. The “rapid deteriorator” trajectory group were the oldest, followed by the “gradual deteriorator” and “late‐deteriorator” groups, similar to that observed with IBM‐FRS.

## 4 DISCUSSION

This study has quantified annual dynamometry‐derived muscle strength and functional status change in a large “real‐world” IBM cohort with long follow‐up duration and also identified distinct subgroups, according to change of grip and knee extension strength and functional status change over time.

Only a small number of previous studies have quantified muscle strength change in IBM cohorts, and have been limited by smaller cohort sizes (N = 11–55), short term follow‐up (maximum 4 years), and limited number of repeated strength measurements.^1^, ^7^, ^8^, ^9^ Furthermore, these studies did not compare strength change across genders. Our study has identified that the pattern and annual rate of strength change differ markedly between male and females.

Strength of all movements in our cohort was lower than reference values; however, this was most pronounced for knee extension and ankle dorsiflexion. These findings are consistent with previous findings. Both Allenbach et al.^4^ and Hogrel et al.^1^ reported the lowest predicted strength of knee extension and ankle dorsiflexion in 22 and 13 participants with IBM, respectively. Our findings, in a larger cohort, help confirm and further quantify the pattern of strength loss in IBM.

Previous reports describe wide variation in the mean annual progression of myometry‐assessed weakness from 3% to 15%.^22^, ^23^ The selective pattern of progression of weakness seen in our cohort in the upper limbs, with predisposition for grip weakness and preservation of wrist extension and elbow flexion, was as expected. In the lower limbs, annual progression was most severe for knee extension, in keeping with previous research describing this as the most affected muscle group in IBM.^4^

In clinical practice, it is evident that not all patients with IBM follow a similar trajectory of disease progression; our study attempted to define whether there is a continuous spectrum of severity or whether patients congregate into discrete subgroups. The variation of progression among trajectory subgroups was marked. Only a minority of study participants were tested for CN1A positivity; the identified association between CN1A positivity and rapid deterioration of grip strength warrants further research in other similar IBM cohorts.

Change in functional status, as measured by the NSS and IBM‐FRS, differed between the genders, with the male cohort demonstrating more rapid deterioration. The observed difference was greatest according to the IBM‐FRS, and may indicate that the observed rapid strength loss in the male cohort translates into more rapid deterioration of functional status. Several previous studies have investigated longitudinal change of functional status in IBM. Hogrel et al. reported a reduction of 22% of the IBM‐FRS over a 48‐month period in their cohort of 13 participants with IBM.^1^ This was in association with a 50% loss of knee extension strength. Our study replicates these findings; however, differing magnitudes of strength and functional status loss may be, in part, due to contrasting methodologies.

Responsive and clinically meaningful outcome measures are crucial to the success of clinical trials. The RESILIENT Study, a recent trial of bimagrumab, an activin type 2 receptor antibody, in 251 participants with IBM did not meet the primary end point of significant change from baseline in the 6‐Minute Walk Distance (6‐MWD) test.^6^ The authors highlighted concerns over suitability of the 6‐MWD test for future trials. The 6‐MWD test has received FDA approval following use in other neuromuscular trials, yet was designed as a sub‐maximal exercise test for study participants with pulmonary and cardiac disease,^24^ and had only been validated in small IBM populations.^25^ Our identification of IBM subgroups, according to longitudinal strength and functional status change, may allow for more focused eligibility criteria for clinical trials reducing baseline heterogeneity among treatment and placebo groups. Variability in baseline strength of the RESILIENT Study was also notable with the mean quadriceps quantitative myometry in the placebo group of 69 newtons but a standard deviation of 72 newtons. Limiting study inclusion to participants with higher than expected strength loss based on an extended screening period of longitudinal assessments may allow for improved probability of detection of treatment efficacy, with prevention of deterioration, rather than improvement of strength, a more appropriate approach.

This study does have several limitations. First, strength values, by means of dynamometry, were collected in a clinical setting, thus potentially introducing inaccuracies due to several less controlled variable factors, such as patient motivation, concurrent co‐morbidities, compliance with advice on regular exercise, and examiner technique. Second, measurement of strength and functional status at differing time points made comparison at set time points more challenging. Third, date of diagnosis was used as the baseline time point; it is possible that date of symptom onset may provide further insights; however, these data were not available, and in the majority of cases detailed monitoring using quantitative muscle testing only took place once the diagnosis was secured. Finally, percentage change from each participant's baseline was calculated, as opposed to the percentage change from predicted “normal values”; although the method we used minimizes baseline strength value variation due to age and gender, this may have led to over‐estimation of change in participants with low baseline strength. Further research is now needed to fully delineate the role of dynamometry‐derived strength in IBM clinical practice and trials. Replication of our work in an independent cohort is required to add credence to our putative identification of IBM subgroups. Dynamometry‐derived strength measurements could be used within an IBM interventional trial, with the aim of investigating the ability to detect change. Furthermore, genetic comparison of these subgroups may provide better understanding of the etiopathogenesis of IBM. This study used both the IBM‐FRS and NSS as measures of function; the IBM‐FRS is a valid method of function assessment in IBM.^15^ However, although the NSS has demonstrated utility in several IBM trials,^14^, ^26^, ^27^ it has not been formally validated in this disease.

In conclusion, our study has quantified muscle strength and functional status change in a large real‐world IBM cohort and identified differences according to gender, age of onset, and possibly CN1A status. We have identified clinical subgroups within the IBM cohort, according to strength and functional status change over time. These findings may lead to enhanced clinical prognostication and potential stratification of trial participants.

## 5 CONFLICT OF INTEREST

None of the authors has any conflict of interest to disclose.

## ETHICAL PUBLICATION STATEMENT

We confirm that we have read the Journal's position on issues involved in ethical publication and affirm that this report is consistent with those guidelines.

## Supporting information


**Figure S1** Knee extension change over time.
