# Supplementary figures and images for: Long‐term strength and functional status in inclusion body myositis and identification of trajectory subgroups
Source: Muscle Nerve. 2020 Mar 13;62(1):76–82. doi: 10.1002/mus.26859 (PMC8629114; doi:10.1002/mus.26859)

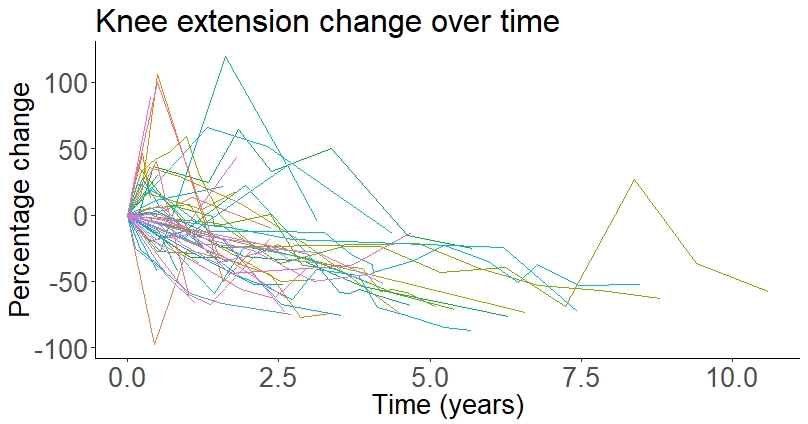

Supplement: Supplementary file 1 — Figure S1 Knee extension change over time. [file MUS-62-76-s001.jpeg]
